# Amplification-free detection of zoonotic viruses using Cas13 and multiple CRISPR RNAs

**DOI:** 10.1099/jgv.0.002169

**Published:** 2025-11-03

**Authors:** Caitlin H. Lamb, Silke Riesle-Sbarbaro, Joseph B. Prescott, Aartjan J. W. te Velthuis, Cameron Myhrvold, Benjamin E. Nilsson-Payant

**Affiliations:** 1 Department of Molecular Biology, Princeton University, Princeton, NJ 08544, USA; 2 Center for Biological Threats and Special Pathogens, Robert Koch Institute, 13353 Berlin , Germany; 3 Department of Chemical and Biological Engineering, Princeton University, Princeton, NJ 08544, USA; 4 Omenn-Darling Bioengineering Institute, Princeton University, Princeton, NJ 08544, USA; 5 Department of Chemistry, Princeton University, Princeton, NJ 08544, USA; 6 TWINCORE, Centre for Experimental and Clinical Infection Research, a joint venture between the Helmholtz Centre for Infection Research and the Hannover Medical School, Hannover, Germany; 7 Cluster of Excellence RESIST (EXC 2155), Hannover Medical School, 30625 Hannover, Germany; 8 Department of Microbiology, Tumor and Cell Biology, Karolinska Institutet, Stockholm, Sweden

**Keywords:** clustered regularly interspaced short palindromic repeats (CRISPR), hantavirus, influenza virus, nucleic acid detection

## Abstract

Zoonotic viruses such as hantaviruses and influenza A viruses present a threat to humans and livestock. There is thus a need for methods that are rapid, sensitive and relatively cheap to detect infections with these pathogens early. Here, we use an amplification-free clustered regularly interspaced short palindromic repeats-associated protein 13 (CRISPR-Cas13)-based assay, which is simple, cheap and field-deployable, to detect the presence or absence of genomic hantavirus or influenza A virus RNA. In addition, we evaluate whether the use of multiple CRISPR RNAs (crRNAs) can improve the sensitivity of this amplification-free method. We demonstrate that for the hantaviruses Tula virus (TULV) and Andes virus (ANDV), a combination of two or three crRNAs provides the best sensitivity for detecting viral RNA, whereas for influenza virus RNA detection, additional crRNAs provide no consistent benefit. We also show that the amplification-free method can be used to detect TULV and ANDV RNA in tissue culture infection samples, ANDV from hamster lung samples and influenza A virus RNA in clinical nasopharyngeal swabs. In clinical samples, the Cas13 assay has an 85% agreement with RT-qPCR for identifying a positive sample. Overall, these findings indicate that amplification-free CRISPR-Cas13 detection of viral RNA has potential as a tool for rapidly detecting zoonotic virus infections.

## Introduction

Zoonotic viruses, such as hantaviruses and influenza viruses, pose serious threats to human health. Hantaviruses are a family of tri-segmented negative-sense RNA viruses that naturally circulate in rodents and other small mammals, where they cause life-long asymptomatic infections [1,2]. Viral particles are shed in the urine and faeces of infected animals, and zoonotic infections of humans can occur by the inhalation of dried or aerosolized secretions. Clinical manifestations of hantavirus infections in humans can range in disease severity. For instance, Old World hantaviruses can cause haemorrhagic fever with renal syndrome or the milder nephropathia epidemica. New World hantaviruses can cause hantavirus cardiopulmonary syndrome (HCPS) with mortality rates of up to 50%. Although up to 200,000 human hantavirus cases are recorded annually, due to the asymptomatic or mild flu-like symptoms in most human infections with Old World hantaviruses, it is likely that actual case numbers are significantly higher [3]. While humans are typically considered to be dead-end hosts for most hantaviruses, rare exceptions exist. For instance, the highly pathogenic Andes virus (ANDV) can transmit between humans through close contact and respiratory secretions. Coupled to their pathogenicity and the ubiquitous presence of rodents and small mammals around human populations, this potential for human-to-human transmission makes hantaviruses like ANDV a significant pandemic and global health threat. Some hantaviruses are mostly non-pathogenic in humans, such as the Old World hantavirus Tula virus (TULV). This makes these viruses key tools for laboratory experiments due to the lack of a BSL-3 requirement.

Influenza A viruses (IAVs) are also single-stranded negative-sense viruses with a segmented genome [4,5]. IAV strains cause seasonal epidemics and occasional pandemics, with disease typically manifesting as a mild to severe respiratory illness. IAV has a wide range of mammalian and avian hosts, such as birds, pigs, cows and humans, which creates many opportunities for zoonotic spillover [5]. Moreover, co-infection of a single host with multiple IAV strains can lead to reassortment, producing genetically novel viruses with pandemic potential. A recent example is the 2009 H1N1 IAV pandemic, which was caused by a spillover event from a pig infected with a triple-reassorted IAV strain [5]. Thus, as with hantaviruses, animal reservoirs play a critical role in the emergence of new IAV strains.

To address the looming threat posed by emerging zoonotic RNA viruses, we rely on fast detection methods for surveillance and early diagnosis. Several molecular tools are available to diagnose and study influenza viruses, including Reverse transcription quantitative PCR (RT-qPCR), primer extension, sequencing and antigen-based techniques. However, the tools to diagnose and study hantaviruses are more limited. Currently, hantavirus detection relies on serology for IgG and IgM, and RT-qPCR [6]. Serology and RT-qPCR are both labour- and time-intensive and require equipment and laboratory infrastructure to be conducted. Given these challenges, additional strategies are needed to detect hantaviruses and emerging IAVs.

**Fig. 1.**
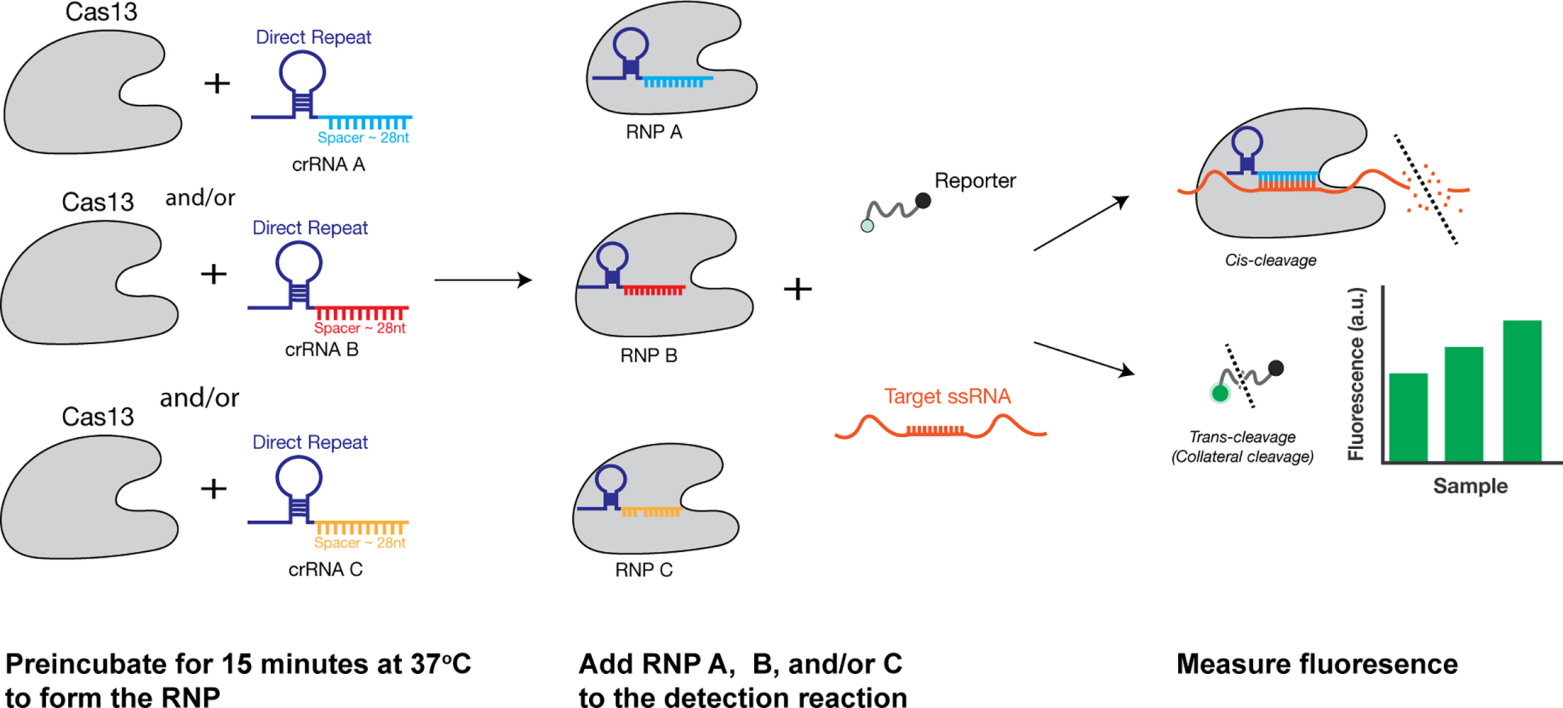
Workflow of multi-crRNA Cas13-based amplification-free detection. Cas13 is first complexed to the crRNA to form the ribonucleoprotein complex [(RNP) A, B or C]. The RNP is then added to the detection reaction containing the fluorescent reporter and target RNA. The Cas13 RNP finds the target RNA, activates and cleaves the reporter in trans, generating a fluorescent signal.

One promising strategy for the surveillance of zoonotic viruses is clustered regularly interspaced short palindromic repeats-associated protein 13 (CRISPR-Cas13)-based nucleic acid detection [7,10]. CRISPR-Cas13-based detection methods harness the *trans*-cleavage activity of the RNA-guided RNase, Cas13, along with an RNA reporter, allowing for enhanced sensitivity and specificity relative to hybridization alone [7,8, 11, 12]. Importantly, these methods can be used to directly detect viral RNA (vRNA) without the need for reverse transcription or nucleic acid amplification [13,15]. Furthermore, it has been shown that using multiple CRISPR RNAs (crRNAs) to target different sequences in the same RNA molecule can increase the sensitivity of severe acute respiratory syndrome coronavirus 2 (SARS-CoV-2) detection (Fig. 1) [13].

Although Cas13 assays that detect hantavirus and IAV RNA using amplification have been reported [10,16], an amplification-free assay would be simpler and easier for rapid screening of suspicious clinical and environmental samples. We previously used an amplification-free Cas13-based method to detect aberrant IAV RNAs [15]. Here, we sought to use amplification-free CRISPR-Cas13 to detect the genetic material of both an Old World and New World hantavirus, TULV and ANDV, respectively, in tissue culture infection samples using one or multiple crRNAs. Furthermore, we also validate this amplification-free method to efficiently detect hantavirus RNA in infected animals. We also explored whether this method can be applied to the detection of IAV genomic RNA. We found that using multiple crRNAs enhances detection sensitivity for both the New World hantavirus ANDV and the Old World hantavirus TULV. However, in the case of IAV, multiple crRNAs did not reliably improve the detection sensitivity. We therefore conclude that amplification-free Cas13 detection using multiple crRNAs has the potential to be a powerful approach to detect zoonotic virus infection, but that it is limited in quantifying the number of genome copies present in the sample and does not always increase the sensitivity of the Cas13 detection and that the assay will need to be optimized for each virus separately.

## Methods

### Preparation of synthetic RNA and primers

Primers were synthesized by Integrated DNA Technologies (IDT) and resuspended in nuclease-free water to 100 μΜ. Primers were stored at −20 °C and were further diluted prior to analysis. crRNAs were synthesized by IDT and resuspended in nuclease-free water to 100 µM. crRNAs listed in Table S1 were stored at −80 °C and were further diluted prior to analysis.

### *In vitro* transcription of target RNA

To generate IAV segment 5 or TULV and ANDV S segment RNA molecules, we first generated PCR products that added a T7 promoter to the 5′ terminus of the vRNA sequence. Specifically, we used a 2× Q5 Master Mix (NEB), 100 ng of a pPolI-IAV-NP, pPolI-ANDV-S or pPolI-TULV-S plasmid and 1 µM of the relevant primers (Table S3). PCR products were analysed by gel electrophoresis before purification using a PCR cleanup kit (NEB). T7 transcriptions were performed using 1 µg DNA template, 5 mM NTPs, 1 mM DTT, 1 U Murine RNase inhibitor (APExBIO), 1× T7 reaction buffer (Search Bioscience) and 100 U NxGen T7 enzyme. Reactions were incubated at 37 °C for ~3 h, treated with DNase for 1 h and then purified using a PCR clean-up kit (Zymo Research).

### Cell culture

Human alveolar basal epithelial carcinoma (A549) cells (ATCC, CCL-185) and African green monkey kidney epithelial (Vero E6) cells (ATCC, CRL-1586) were commercially obtained. All cells were routinely screened for mycoplasma and grown in Dulbecco’s modified Eagle medium (DMEM) containing pyruvate, high glucose and l-glutamine (GeneDepot) supplemented with 10% FBS (Gibco) and penicillin and streptomycin at 37 °C and 5% CO_2_.

### Virus culture and titration

ANDV (*Orthohantavirus andasense*) strain Chile-9717869 was a kind gift by Piet Maes (KU Leuven, Belgium). TULV (*Orthohantavirus tulaense*) strain Moravia/Ma5302v/94 was a kind gift by Rainer Ulrich (Friedrich-Loeffler-Institute, Germany). Virus propagation and titration was performed as previously described [17]. In brief, ANDV and TULV were propagated in Vero E6 cells in DMEM supplemented with 2% FBS, l-glutamine, Minimum Essential Medium (MEM), non-essential aa and penicillin and streptomycin. During virus propagation, cell culture supernatants containing infectious virus particles were collected between 5 and 14 days post-infection and replaced with fresh cell culture media. Pooled viral stocks were cleared from cellular debris by centrifugation (4,000 ***g***, 10 min, 4 °C) prior to three buffer exchanges in PBS and concentration using Amicon Ultra-15 centrifugal filter units (100 kDa molecular weight cut-off).

Concentrated viruses were titrated by immuno-plaque assays in Vero E6 cells. Briefly, Vero E6 cells were infected with sequential tenfold dilutions of virus and overlayed with 1.2% Avicel in MEM containing 2% FBS, HEPES, MEM non-essential aa and penicillin and streptomycin. At 7 days post-infection, cells were fixed with 3.7% formaldehyde, permeabilized with 0.5% Triton X-100, blocked with 5% BSA in PBS and immunostained with cross-reactive primary monoclonal antibody targeting TULV NP (TULV1 [18]) and an IRDye-conjugated anti-mouse IgG (IRDye 800CW, 926-32210) secondary antibody. Near-infrared fluorescence signal of stained plaques was detected using an Odyssey CLx imaging system (LI-COR) and analysed with the Image Studio software (LI-COR).

### Cell culture infections with hantaviruses

All cell culture work involving live ANDV was performed in the BSL-3 facility of the Hannover Medical School according to institutional biosafety requirements. For infections, ~2.5×10^5^ A549 cells were infected with TULV or ANDV at an m.o.i. of 0.1 in DMEM supplemented with 2% FBS, l-glutamine, MEM non-essential aa and penicillin and streptomycin. At the indicated time points, cell culture supernatants were removed, and cells were washed once with PBS. Cells were lysed and viruses were inactivated in TRIzol reagent (Invitrogen) or RIPA buffer supplemented with 1% SDS.

### RNA sample preparation

IAV clinical sample RNA extraction was carried out using TRI reagent (Molecular Research Centre, Inc.) following the manufacturer’s instructions and as described previously [15,19]. Total RNA from hantavirus-infected cells or tissues was extracted using TRIzol reagent (Invitrogen) and the Direct-Zol RNA Miniprep Kit (Zymo Research) according to the manufacturers’ instructions. The RNA concentration was determined using a NanoDrop One spectrophotometer (Thermo Fisher) and diluted in RNase-free water prior to analysis.

### Reverse transcription quantitative PCR

Reverse transcription of RNA samples was performed using oligo(dT)/random hexamer primers and the PrimeScript RT Master Mix (TaKaRa Bio) according to the manufacturer’s instructions. Quantitative real-time PCR was performed using the TB Green Premix Ex Taq II (Tli RNase H Plus) Master Mix (TaKaRa Bio) on a LightCycler 480 Instrument II (Roche) according to the manufacturers’ instructions. Primers targeting human GAPDH (for: GAAGGTGAAGGTCGGAGTC; rev: GAAGATGGTGATGGGATTTC) and previously described universal [20] S segment hantavirus primers (for: CAGGAYATGVGRAAYACVATHATGGC; rev: CTCWGGRTCCATRTCATCMCC) were used. Delta-delta-cycle threshold (ΔΔCT) values were determined relative to the housekeeping gene GAPDH and normalized to the mean of the uninfected control group. Error bars indicate the sd from three biological replicates (*n*=3).

### Western blot

For western blot analysis, whole-cell lysates were obtained through cell lysis in RIPA buffer containing 1% SDS and sonication. Lysates were analysed by SDS-PAGE and transferred onto nitrocellulose membranes. Membranes were stained with an actin antibody (Invitrogen; MA5-11869) and a previously described Puumala virus NP antibody (clone 5E11) that is cross-reactive against both ANDV and TULV NP (a kind gift by Indrė Kučinskaitė-Kodzė, Vilnius University). Primary antibody staining was visualized using IRDye-conjugated secondary antibodies (IRDye 680RD, 926-68070; IRDye 800CW, 926-32210). Near-infrared fluorescent signalling was imaged using an Odyssey CLx imaging system (LI-COR) and analysed with the Image Studio software (LI-COR).

### Cas13-based detection reactions

Each reaction contained 10 nM LbuCas13a, 4 mM HEPES pH 8.0, 12 mM KCl and 1% PEG, 2 U µl^−1^ RNAse inhibitor murine (New England Biolabs), 0.25 µM 6UFAM (/FAM/UUUUUU/3IABKFQ/), 14 mM MgOAc, 5 nM crRNA (Table S1) and the reported amount of target RNA. First, LbuCas13a was combined with crRNA at a ratio of 2 : 1. The crRNA-Lbucas13a complex was subsequently added to a Master Mix containing HEPES, KCl, PEG, RNAse inhibitor and MgOAc for a final volume of 44 µl in 96-well plates. After mixing, 20 µl was transferred in duplicate to 384-well plates. The plate was then sealed and placed in a BioTek Synergy H1 Plate Reader and incubated at 37 °C for 3 h. Fluorescence was measured every 5 min. Alternatively, 20 µl was transferred in duplicate to 96-well plates, which were sealed and placed in a LightCycler 480 Instrument (Roche). Plates were incubated at 37 °C for 3 h and fluorescence was measured every 2 min.

### Animals and husbandry

Fifteen 4–5-week-old female Syrian hamsters were purchased from Janvier labs, France, transported to the BSL-4 facility of the Robert Koch Institute and randomly assigned to three groups of five animals each. A maximum of four hamsters was housed in IVC GR900 cages (900 cm^2^, Tecniplast Sealsafe). Low-dust bedding, a shelter and nesting material were provided. Animals were acclimated for 7 days and kept in a reversed 12-h daylight cycle, at 21 °C (±1 °C) and 50% (±10%) of relative humidity. Water and food were offered *ad libitum* throughout the experiment.

### *In vivo* infection and sample processing

All animal work with infectious ANDV was performed in the BSL-4 laboratory at the Robert Koch Institute. Under isoflurane anaesthesia, hamsters were weighed, ear-tagged and intranasally inoculated with either 20, 200 or 2,000 f.f.u. (*n*=5 for each group) of ANDV Chile-9717869 in 100 µl of sterile PBS using a p200 pipette. Animals were scored and observed daily for the development of signs of disease. Animals that reached the endpoint scoring criteria were euthanized using an overdose of isoflurane and exsanguination by cardiac puncture. Sections of lung tissues were collected, weighed and homogenized in TRIzol, in a volume ratio of 1/20, using a stainless-steel bead and a TissueLyser II (QIAGEN) homogenizer and stored at −80 °C.

### Clinical samples

As previously described [15], nasopharyngeal samples were taken during routine testing from patients hospitalized at Addenbrookes Hospital during the 2016–2017, 2017–2018, 2018–2019 and 2019–2020 flu seasons. The study protocol was reviewed and approved by the Health Research Authority (IRAS ID 258438; REC reference 19/EE/0049). Patients were positive for either H1N1 or H3N2, but not other respiratory viruses, and samples were taken from a range of pathologies (asymptomatic to death). Per sample (typically 1.5 ml), 250 µl was used for total RNA extraction using TRI Reagent. RNA was dissolved in 10 µl RNase-free water and stored at −70 °C prior to Cas13 analysis.

### Statistical testing

GraphPad Prism 10 software was used for statistical testing. Unless otherwise stated, error bars represent standard deviation and individual data points indicate biological repeats.

## Results

### Combining crRNAs can enhance hantavirus detection

Currently, there are limited tools and methods to detect and study hantaviruses. As previously shown for IAV, reverse transcription PCR or RT-qPCR may introduce sequence-specific amplification biases [15]. In addition, these methods require the end user to optimize temperature, primer design and probe design. Here, we used amplification-free, CRISPR-Cas13-based RNA detection for the identification of hantavirus genomic RNA. We used the Cas13a orthologue from *Leptotrichia buccalis* (LbuCas13a) since we and others previously have shown that this orthologue has the highest sensitivity and activity compared to other Cas13 orthologues [15,21].

Using Activity-informed Design with All-inclusive Patrolling of Targets (ADAPT), a system for the automated design of crRNAs [22], we designed crRNAs against the vRNA of the TULV or ANDV small (S) segment, which is the most abundantly expressed segment during hantavirus infections. We picked the top three ADAPT-designed crRNAs (named A, B and C) (Table S1) based on the highest ADAPT-predicted activity. We then tested these three crRNAs individually, in pairwise combinations or all together against *in vitro* transcribed (IVT) full-length S segment ANDV or TULV vRNA molecules.

**Fig. 2.**
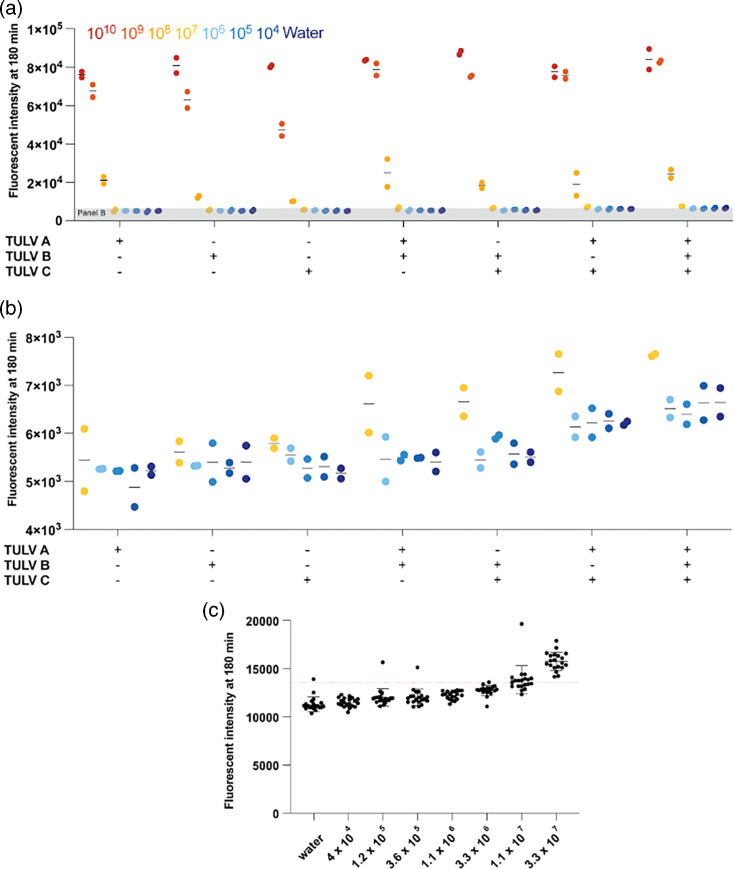
Validation of multi-crRNA Cas13-based amplification-free detection of TULV S segment vRNA using IVT S segment vRNA. (**a**) Detection of a 10-fold dilution series of IVT vRNA-sense S segment TULV RNA using an amplification-free Cas13-based approach using one, two or three crRNAs. (**b**) A subset of data from A. Each point represents one technical replicate. There are two technical replicates. (**c**) Threefold dilution series to determine the limit of detection. The red line indicates our limit of detection cut-off of 3 sd above the water input control. Each point represents one technical replicate. There are 20 technical replicates. The mean and sd are shown.

For TULV, we observed that when used individually, all three crRNAs yielded a signal at least 3 sd above the water control when 10^8^ copies/μl of IVT target RNA were present in the sample. Unless defined otherwise, we used this cut-off of 3 sd to define whether our assay had produced a statistically significant increase relative to the negative control. TULV crRNA A showed the highest fluorescent signal at 10^8^ copies/µl, followed by TULV crRNAs B and C (Fig. 2a, b). Combining two crRNAs increased the signal compared to the individual TULV crRNAs, allowing detection down to ~10^7^ copies/μl of the IVT target RNA when technical duplicates were analysed (Fig. 2a, b). This detection signal above background was higher for all pairwise combinations of crRNAs compared to the individual crRNAs. Furthermore, combining three TULV crRNAs resulted in a higher and a less variable signal (Fig. 2a, b). Thus, by combining all three crRNAs when detecting TULV vRNA, LbuCas13a can achieve the best performance.

For diagnostic use, a limit of detection (LOD) analysis based on many technical replicates is critical. To determine the LOD for the triple crRNA set, which we defined as the point at which 19 out of 20 technical replicates were above background, we repeated the LbuCas13a assay 20 times using serial dilutions of different concentrations of synthetic target RNA as input. Starting at 3.3×10^7^ copies/μl of IVT target RNA, we generated a threefold dilution series and measured each concentration using 20 technical replicates. We determined the LOD for TULV to be 3.3×10^7^ copies/μl IVT RNA (Fig. 2c). Furthermore, we validated the specificity of the crRNA set by performing the reaction against IVT ANDV RNA (Fig. S1A, available in the online Supplementary Material). The TULV crRNA set showed little cross-reactivity even at 10^9^ copies/μl IVT ANDV RNA (Fig. S1A).

**Fig. 3.**
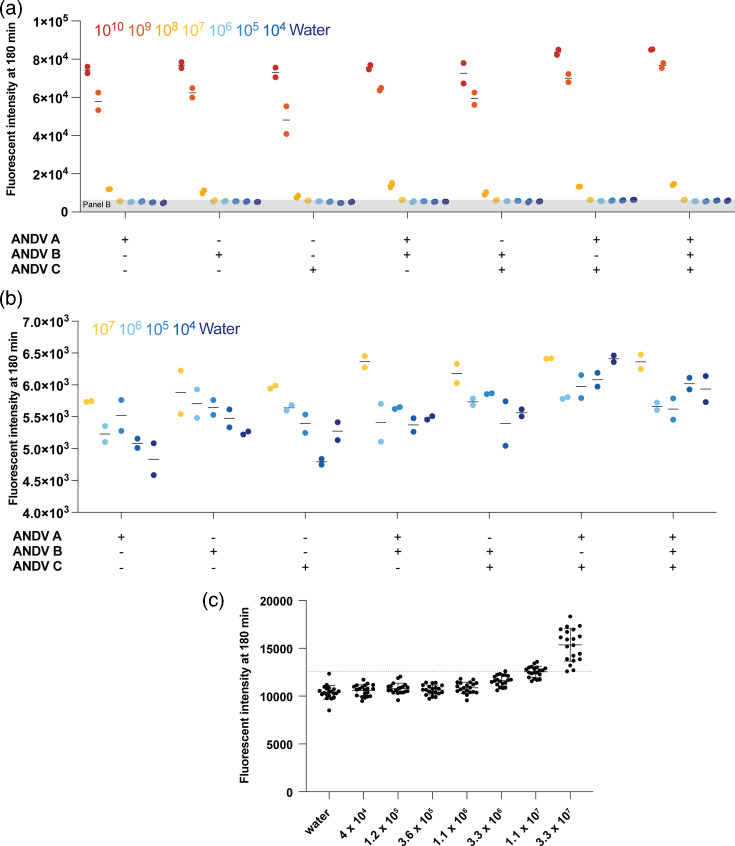
Validation of multi-crRNA Cas13-based amplification-free detection of ANDV S segment vRNA using IVT S segment vRNA. (**a**) Detection of a tenfold dilution series of vRNA-sense S segment IVT ANDV RNA using an amplification-free Cas13-based approach using one, two or three crRNAs. (**b**) Subset of data from (a). Each point represents one technical replicate. There are two technical replicates. (**c**) Threefold dilution series to determine the LOD. The red line indicates our LOD cut-off, 3 sd above the water background. Each point represents one technical replicate. There are 20 technical replicates. The mean and sd are shown.

When we used the ANDV crRNAs, the individual crRNAs A and B yielded a significant signal above background at 10^8^ copies/μl of target RNA, whereas crRNA C was limited to 10^9^ copies/μl of target RNA (Fig. 3a, b). Combining two ANDV crRNAs increased the signal, allowing for detection down to 10^7^ copies/μl of target RNA when we combined crRNAs A and B or B and C, with the combination of crRNAs A and B producing the best signal (Fig. 3a, b). However, combining A and C did not improve target RNA detection. When we combined all three ANDV crRNAs, we did not see a further improvement in sensitivity, keeping the detection at 10^7^ copies/μl RNA per sample (Fig. 3a, b). Taken together, we conclude that combining ANDV crRNAs A and B produced the highest signal above background. As with TULV, we next generated a threefold dilution series and used this series for 20 technical replicates to determine the LOD. We found that the LOD for crRNAs A and B was 3.3×10^7^ copies/μl target RNA for 19 out of 20 technical replicates (Fig. 3c). Finally, we validated the specificity of the guide set by targeting the IVT TULV RNA (Fig. S1B). The guide set shows little cross-reactivity at 10^10^ copies/μl IVT RNA (Fig. S1B).

### Cas13-based detection of hantavirus RNA captures infection dynamics

After determining the LOD, we tested the ability of Cas13 to detect hantavirus vRNA in infected cells. To this end, we infected human lung adenocarcinoma A549 cells, a typical cell line representing the primary site of hantavirus infection in humans, at an m.o.i. of 0.1 with TULV or ANDV and extracted total RNA from cells at 6, 24 and 48 h post-infection (hpi). We next proceeded to detect the S segment vRNA using Cas13 and RT-qPCR.

**Fig. 4.**
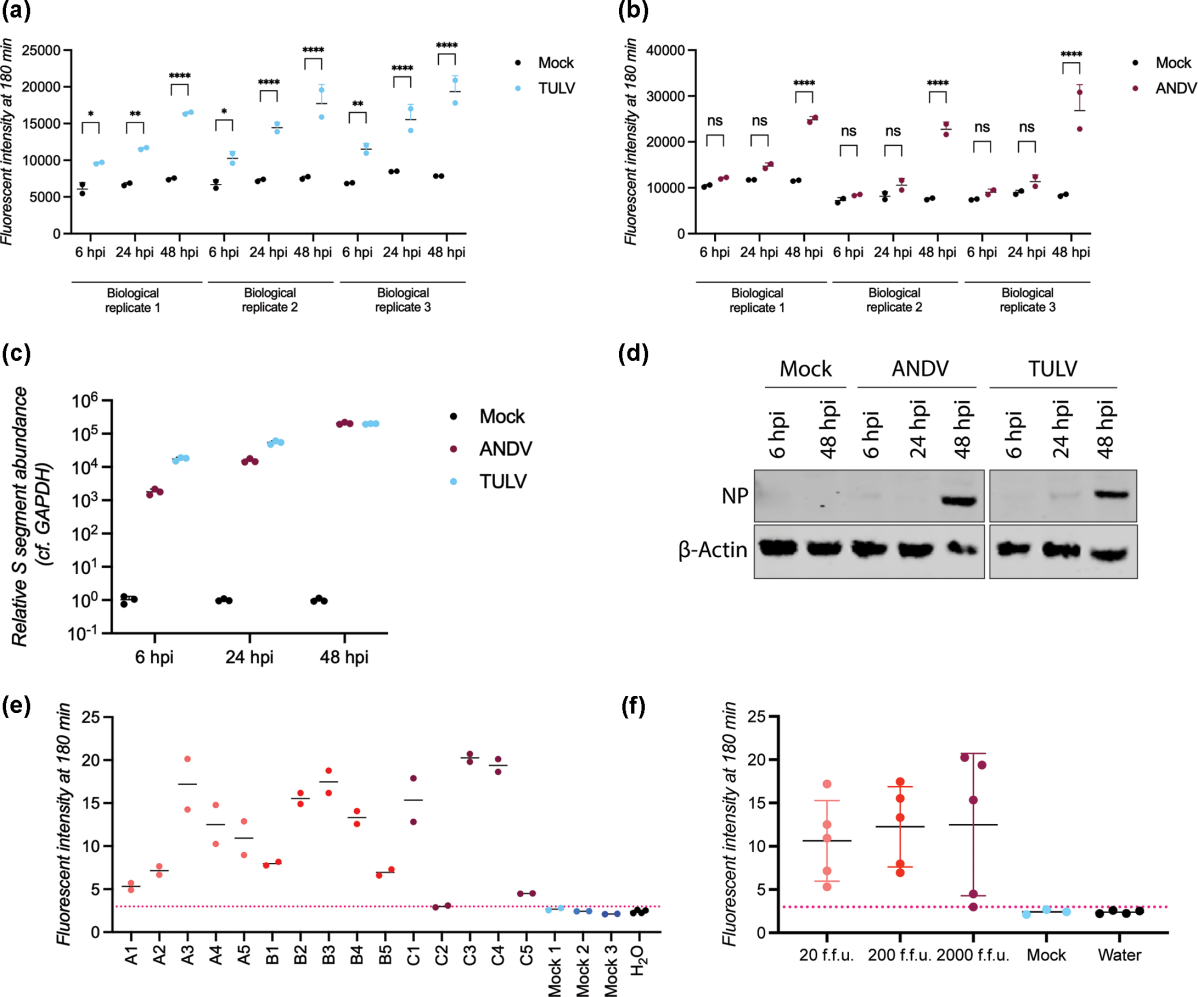
Validation of Cas13-based amplification-free detection of ANDV and TULV in infected cells and animals. Detection of S segment vRNA in A549s infected with (**a**) TULV or (**b**) ANDV at an m.o.i.=0.1 using multi-crRNA Cas13-based amplification-free detection or (**c**) RT-qPCR. (**d**) A549 infected with TULV or ANDV at an m.o.i.=0.1 were analysed by western blot using a pan-hantavirus NP and actin antibody. (**e**) Syrian hamsters were intranasally infected with 20 f.f.u. (A1–A5), 200 f.f.u. (B1–B5) or 2000 f.f.u. (C1–C5) of ANDV (*n*=5 for each group, uninfected control animals *n*=3). At the endpoint of infections, RNA from lung tissues was analysed by CRISPR-Cas13 using a single ANDV S vRNA-targeting crRNA. The dotted line represents 3 sd above the average of the water control. The graphs depicting the CRISPR-Cas13 data (**a, b, e**) show one biological replicate with two technical replicates, represented as points. The graph depicting the CRISPR-Cas13 data (**f**) shows five biological replicates (three for the negative control) represented as points. The graph for RT-qPCR (**c**) represents the mean relative fold change of S segment RNA compared to a housekeeping gene (GAPDH) of three independent biological replicates. For (**a**) and (**b**), two-way ANOVA followed by Šidák’s multiple comparisons test was performed. Significance is indicated as *P<0.05, **P<0.01, ***P<0.001, ****P<0.0001 or ns if not significant.

Using Cas13, the TULV S segment vRNA was first detected at 6 hpi, and its signal showed a more gradual increase towards 48 hpi (Fig. 4a). However, ANDV S segment vRNA was first detected at 24 hpi and its signal increased dramatically at 48 hpi (Fig. 4b). Comparing these Cas13 results with the RT-qPCR readout, we found that the ANDV S segment RNA was detectable at 6 hpi by RT-qPCR (Fig. 4c). In agreement with the Cas13 data, the vRNA signal increased dramatically at 24 and 48 hpi (Fig. 4b, c). The TULV vRNA was first detected at 6 hpi by RT-qPCR and increased steadily at 24 and 48 hpi, similar to what we observed using Cas13 detection (Fig. 4a, c). It is important to note here that the RT-qPCR was not specific to the vRNA molecules in the infection samples, since the RT step was performed using random hexamers. Thus, hantavirus cRNA and mRNA molecules had also contributed to the signal. This key difference between the Cas13 and RT-qPCR assays, combined with the amplification step in the qPCR assay, likely increased the apparent difference in sensitivity. To confirm that the differences in Cas13 and RT-qPCR signals were correlated with differences in ANDV and TULV growth kinetics, we performed western blots. We found a strong viral nucleoprotein (NP) expression at 48 hpi for ANDV and a TULV NP signal at 24 hpi (Fig. 4d). Thus, the CRISPR-Cas13 method provides data that capture information on the viral infection cycle and that agree with traditional molecular methods, even if the sensitivity of the assay is inherently lower due to the lack of an amplification step.

### Cas13 can be used to detect ANDV RNA in infected animals

Having established that we can use our CRISPR-Cas13 method to accurately detect hantavirus vRNA in infected cells, we next sought to validate whether this approach could be used to detect infections in a more biologically relevant model. Since human ANDV and TULV cases – as opposed to some other hantaviruses – are relatively rare or hard to come by, we used a Syrian hamster model for ANDV to test our method. Syrian hamsters not only represent a valid animal model which sustains high levels of viral replication for many hantaviruses, but they are also the only non-primate animal model which results in lethal disease mimicking lethal HCPS-like human disease in animals [23].

Here, Syrian hamsters were intranasally infected with different infectious doses of ANDV, and lung tissue samples were collected at the endpoint of infections. Using a single ANDV S vRNA targeting crRNA, we were able to detect vRNA above 3 sd of background levels using CRISPR-Cas13 in all animals (Fig. 4e). Interestingly, when grouping animals according to the infectious dose of ANDV they received, we found slight differences in fluorescent levels, including a slight increase in vRNA detection at higher infectious doses (Fig. 4f). Overall, however, these data suggest that we can reliably detect ANDV vRNA in a biologically and clinically relevant animal model using our amplification-free CRISPR-Cas13 method.

### Combining crRNAs does not seem to consistently improve IAV detection

Our above results show that using multiple crRNAs can increase the fluorescent signal and LOD of our LbuCas13a assay for detecting hantavirus RNA. To determine the general applicability of using multiple crRNAs for RNA detection, we tested whether it could be used for detecting IAV genomic RNA. To this end, we designed crRNAs targeting IAV vRNA or the replication intermediate, the cRNA, using ADAPT. We picked two crRNAs targeting the IAV vRNA (A and B) and three crRNAs (A, B and C) (Table S1) targeting the cRNA based on the highest ADAPT-predicted activity.

**Fig. 5.**
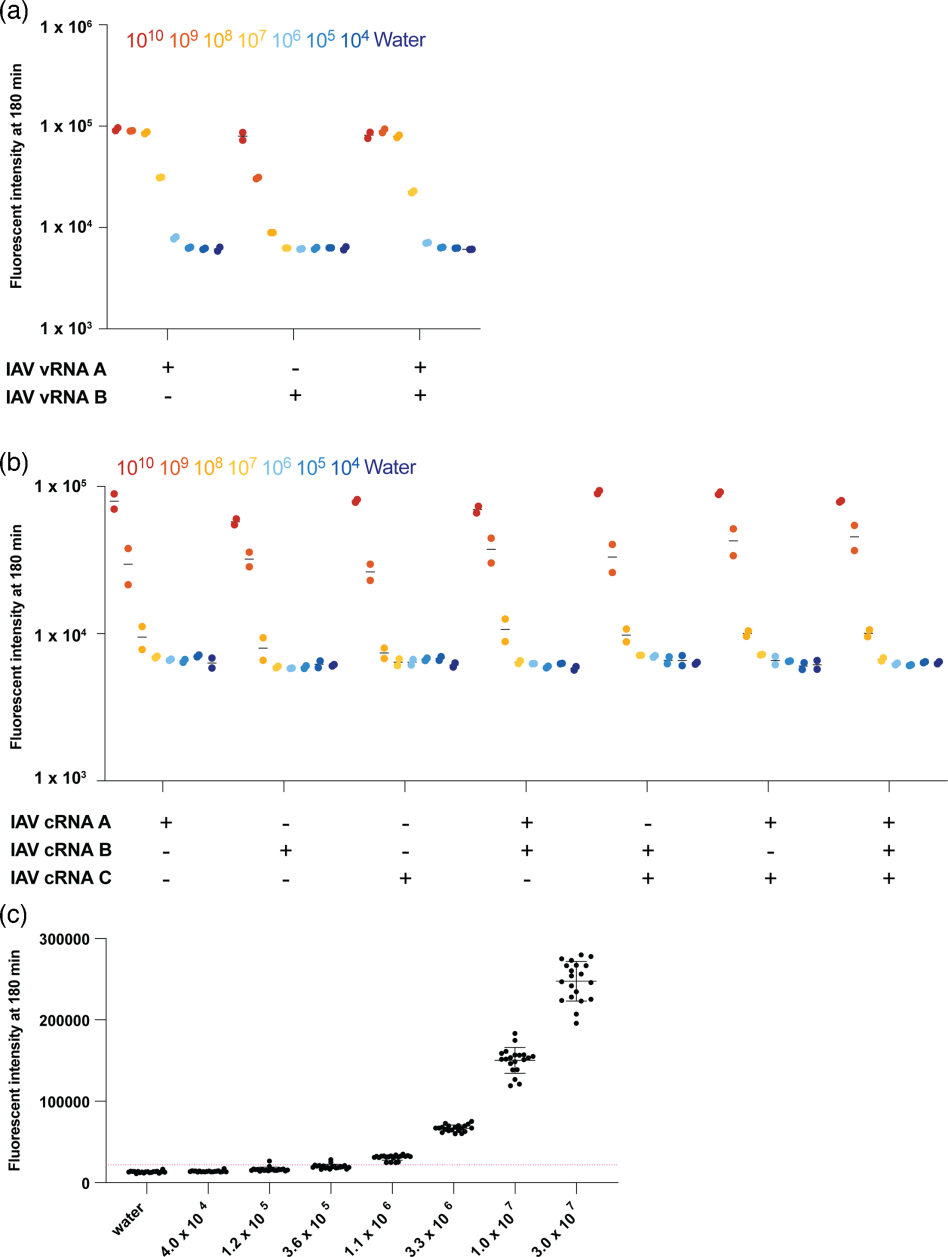
Validation of multi-crRNA Cas13-based amplification-free detection of vRNA- and cRNA-sense IAV segment 5 using IVT vRNA- or cRNA-sense segment 5 RNA. Detection of a tenfold dilution series of (**a**) vRNA-sense or (**b**) cRNA-sense segment 5 IVT IAV RNA using an amplification-free Cas13-based approach using one, two or three crRNAs. Each point represents one technical replicate. There are two technical replicates. (**c**) Threefold dilution series to determine the LOD. The dotted line indicates our LOD cut-off, 3 sd above the no-input control. Each point represents one technical replicate (*n*=20). The mean along with the sd is shown.

We first tested our set of crRNAs against a tenfold dilution series of IVT segment 5 vRNA (Fig. 5a). We found that IAV crRNA A supported a signal above background at 10^7^ vRNA copies/μl sample, while crRNA B only yielded a signal above background at 10^8^ copies/μl sample (Fig. 5a). Combining the two crRNAs did not improve the overall detection of IAV vRNA (Fig. 5a). We next tested our IAV crRNA set targeting the segment 5 cRNA against a tenfold dilution series, starting at 10^10^ copies cRNA/μl sample (Fig. 5b). All three crRNAs were able to produce a signal above background at 10^9^ copies/μl sample. When we combined two or three crRNAs, the assay yielded a signal above background at 10^8^ cRNA copies/μl sample (Fig. 5b), indicating that combining crRNAs targeting the IAV cRNA does improve the detection.

We determined the LOD for the vRNA- and cRNA-targeting crRNAs using a threefold dilution series, starting at 3.0×10^7^ copies/μl, with 20 technical replicates at each concentration. We determined that the LOD of the vRNA-targeting crRNA A was 1.1×10^6^ RNA copies/μl of the IVT target RNA (Fig. 5c). However, we were not able to detect a signal above the background for any combination of cRNA-targeting crRNAs and therefore did not determine an LOD for these crRNAs (Fig. S2).

Finally, we tested whether our vRNA assay was specific to our strain of interest, WSN. We found that our assay was only able to detect A/Brisbane/59/07 (H1N1) and A/Oklahoma/05 (H3N2) at 10^10^ copies/μl sample, but not lower, and unable to detect A/Vietnam/1203/04 (H5N1) at all, indicating that the LOD for the correct IAV sequence is ~3 orders of magnitude lower than the detection of incorrect IAV RNA and thus very specific (Fig. S3).

### Cas13 can be used to detect IAV RNA in clinical samples

We next evaluated whether our IAV crRNAs could be used to diagnose clinical influenza samples. In addition to our vRNA-targeting crRNAs, we also tested the cRNA-targeting crRNAs. cRNA molecules are typically not present in IAV virions but are present in infected cells. We thus reasoned that we could use the vRNA- and cRNA-specific assays to get an indication of the amount of viral and cellular material in the clinical samples. We had previously already shown that some 5S rRNA is present in IAV clinical nasopharyngeal swabs.

**Fig. 6.**
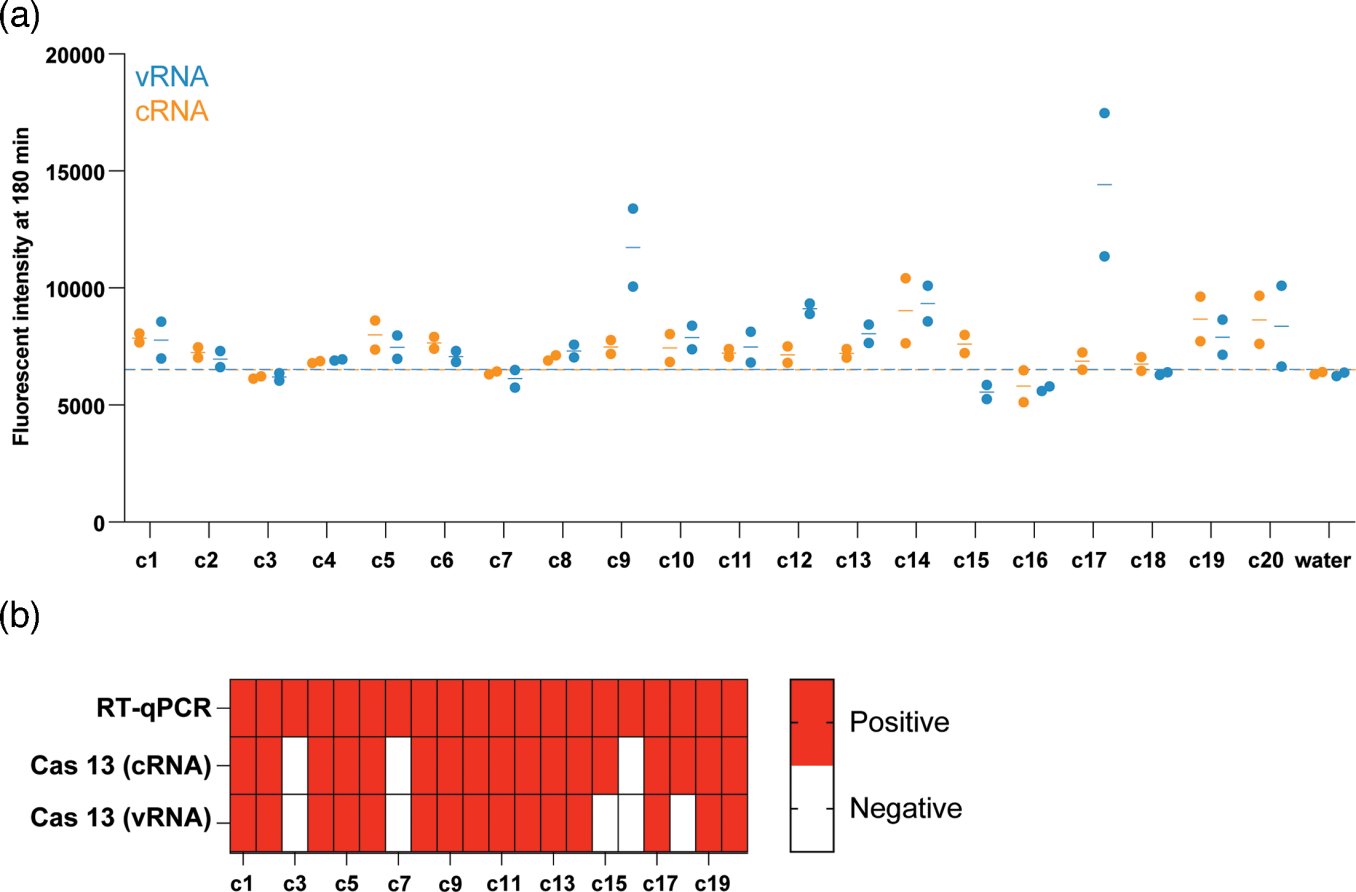
Cas13-based amplification-free detection of vRNA- and cRNA-sense IAV segment 5 in RT-qPCR H1- or H3-positive patient samples. (**a**) IAV segment 5 detection in RT-qPCR H1- or H3-positive patient samples (Table S2). The orange points represent fluorescent values obtained from the Cas13-based assay for cRNA detection, and blue represents fluorescent values obtained for vRNA detection. Dashed lines indicate cut-off for positive detection, determined to be 2 sd above the no-input control. Each point represents one technical replicate. There are two technical replicates for each sample. (**b**) Comparison of the positive or negative diagnosis for each clinical sample based on the RT-qPCR data or the Cas13-based assays.

To test the clinical samples, we extracted total RNA from 20 clinical nasopharyngeal swab samples from patients that had tested positive for seasonal H1N1 or H3N2 using RT-qPCR. These samples included both male and female patients with ages ranging from 1 to 94 years of age (Table S2). We determined which samples were positive based on the fluorescence signal being 2 sd above the mean of the no-input control samples (dashed line). Our vRNA-targeting assay detected IAV RNA in 15 out of 20 positive samples, i.e. 75%, and our cRNA-targeting assay detected IAV RNA in 17 out of 20 samples, i.e. 85% (Table S2, Fig. 6). The cRNA signal was generally lower than the vRNA signal, suggesting that the nasopharyngeal swabs mostly contained viral and limited cellular material. Overall, our results demonstrate that our Cas13-based assay can be used to detect IAV RNA in clinical nasopharyngeal swabs.

## Discussion

Multiple amplification-based vRNA detection methods are available, including RT-qPCR, RPA, and CRISPR-Cas13 and CRISPR-Cas12 approaches [24,26]. Amplification increases the sensitivity of detection and enables more exact quantification, but these characteristics are not always required when a simple yes/no answer suffices. An example is a lateral flow-based determination of SARS-CoV-2 infection. We here evaluated the effectiveness of an amplification-free, CRISPR-Cas13-based detection method for detecting RNA from zoonotic viruses such as hantaviruses and IAV. Our results demonstrate that combining multiple crRNAs can, in some cases, increase detection in agreement with previous SARS-CoV-2 data [13]. However, we also observed that additional crRNAs do not always lead to improvement and that their impact depends on both the target and the crRNA sequence, as the secondary structure of the target can influence the sensitivity of the Cas13 assay. Careful optimization of new CRISPR-Cas13 assays is therefore recommended.

For TULV and ANDV, combining two or three crRNAs enhanced the detection signal compared to single-crRNA conditions. Additionally, we showed that this method can be used to track ANDV and TULV dynamics during infection in tissue culture and that the generated Cas13 data are consistent with RT-qPCR data, meaning that Cas13, although less sensitive than RT-qPCR due to the lack of an amplification step, can still capture relevant trends. Importantly, we acknowledge that this high LOD may cause difficulties in detecting hantaviruses from patient samples. However, our method provides an important step towards a faster and direct method to detect hantaviruses. Furthermore, this method may still be used in environmental settings and detecting hantaviruses in rodent reservoirs, which have been shown to have several orders of magnitude higher viral load. Here, we provide a proof-of-concept for this, and we successfully detected ANDV in infected Syrian hamsters, which are the only non-primate animal model that mimics human HCPS disease (Fig. 4e, f).

In contrast to the hantavirus assay, the use of multiple crRNAs for IAV detection did not significantly enhance detection. For IVT RNA detection, a single vRNA-sense crRNA was the most effective, outperforming additional crRNAs even when combined. Similarly, the cRNA-sense crRNAs showed minimal improvement in signal when used in combination, and the overall signal remained weak compared to vRNA-sense detection, which is to be expected due to much lower levels of cRNA present in infections. Even though a single crRNA provided the best Cas13 sensitivity for segment 5 vRNA, we were able to detect IAV RNA in clinical samples using both the vRNA-sense and cRNA-sense crRNAs.

In summary, while combining multiple crRNAs can improve detection for certain targets, such as hantaviruses, this approach does not translate to enhanced sensitivity across all viruses. Therefore, optimization must be tailored to the specific virus, segment and crRNA set being tested. However, this optimization burden is relatively limited in light of the simplicity of the assay and its potential as a rapid diagnosis tool.

## Supplementary material

10.1099/jgv.0.002169 Uncited Supplementary Material 1.

## Abbreviations

ADAPT: Activity-informed Design with All-inclusive Patrolling of Targets
ANDV: Andes virus
Cas13: CRISPR-associated protein 13
CRISPR: clustered regularly interspaced short palindromic repeats
crRNA: CRISPR RNA
DMEM: Dulbecco’s modified Eagle medium
HCPS: hantavirus cardiopulmonary syndrome
hpi: hours post-infection
IAV: influenza A virus
IDT: Integrated DNA Technologies
IVT: *in vitro* transcribed
LOD: limit of detection
MEM: minimum essential medium
RT-qPCR: reverse transcription quantitative PCR
SARS-CoV-2: severe acute respiratory syndrome coronavirus 2
TULV: Tula virus
vRNA: viral RNA
