# Supplementary material for: Amplification-free detection of zoonotic viruses using Cas13 and multiple CRISPR RNAs
Source: J Gen Virol. 2025 Nov 3;106(11):002169. doi: 10.1099/jgv.0.002169 (PMC12582395; doi:10.1099/jgv.0.002169)
Supplement: Uncited Supplementary Material 1. [file jgv-106-02169-s001.pdf]

Supplemental Information

Amplification-free Detection of Zoonotic Viruses Using Cas13 and Multiple CRISPR RNAs

Caitlin H. Lamb[1], Silke Riesle-Sbarbaro[2], Joseph B. Prescott[2], Aartjan J. W. te Velthuis[1,*], Cameron Myhrvold[1,3,4,5,*], Benjamin E. Nilsson-Payant[6,7,8*]

[1] Department of Molecular Biology, Princeton University, Princeton, NJ 08544

[2] Center for Biological Threats and Special Pathogens, Robert Koch Institute, 13353 Berlin, Germany

[3] Department of Chemical and Biological Engineering, Princeton University, Princeton, NJ 08544, USA

[4] Omenn-Darling Bioengineering Institute, Princeton University, Princeton, NJ 08544, USA

[5] Department of Chemistry, Princeton University, Princeton, NJ 08544, USA

[6] TWINCORE, Centre for Experimental and Clinical Infection Research, a joint venture between the Helmholtz Centre for Infection Research and the Hannover Medical School, Hannover, Germany

[7] Cluster of Excellence RESIST (EXC 2155), Hannover Medical School, Carl-Neuberg-Straße 1, 30625 Hannover, Germany

[8] Department of Microbiology, Tumor and Cell Biology, Karolinska Institutet, Stockholm, Sweden

* address correspondence to: A.J.W.t.V. (aj.te.velthuis@princeton.edu); C.M. (cmyhrvol@princeton.edu); or B.E.N (benjamin.nilssonpayant@ki.se)

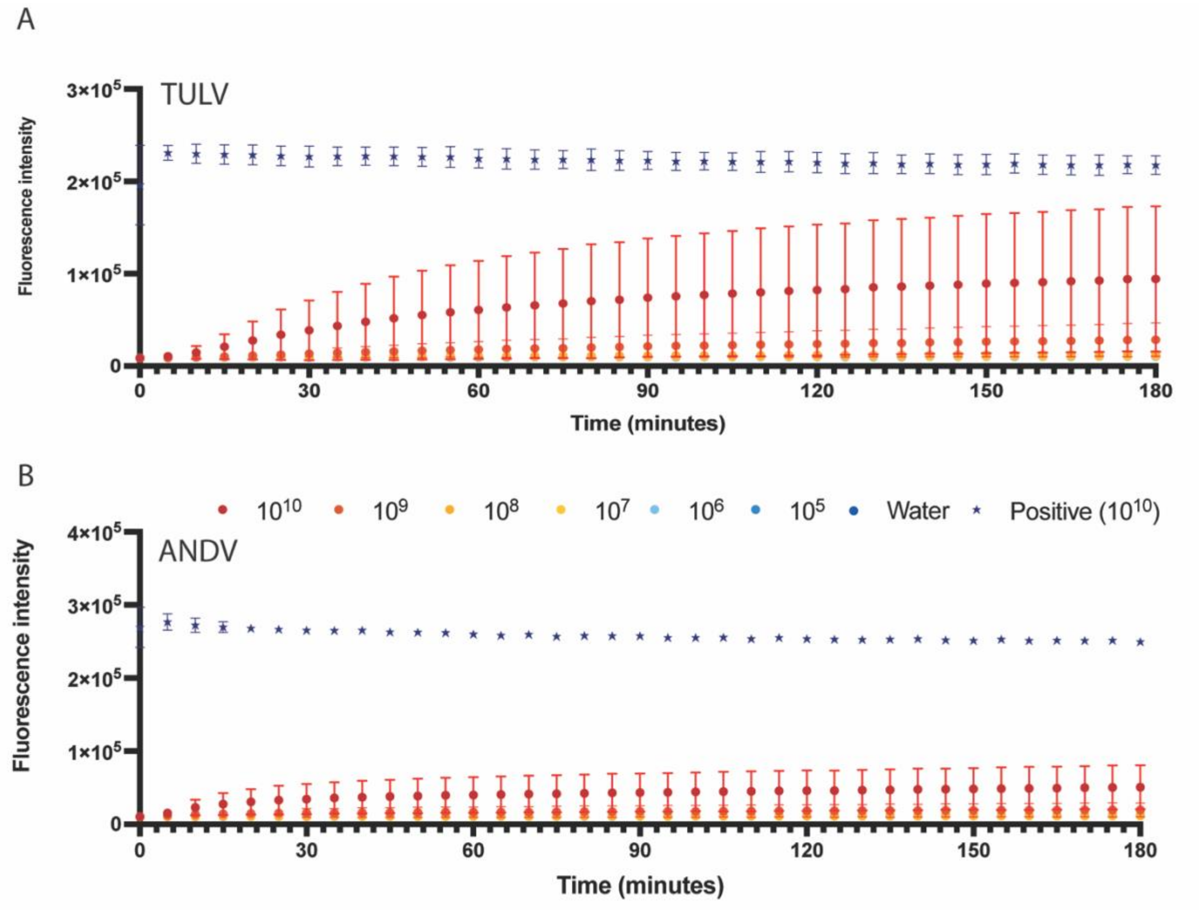

Figure S1. Determining the specificity of TULV and ANDV crRNA sets. 10-fold dilution series starting from 10^{10} RNA molecules/µl to determine cross reactivity of TULV and ANDV crRNA sets. (A) The TULV crRNA set from Fig. 2 was used to target IVT ANDV RNA and (B) the ANDV crRNA set from Fig. 3 was used to target IVT TULV RNA. 10^{10} IVT ANDV RNA molecules/µl or 10^{10} IVT TULV RNA molecules/ µl were used as positive controls for the ANDV crRNA or TULV crRNA sets, respectively. The kinetic curves are shown. Each point represents the mean of technical duplicates and error bars indicate the standard deviation.

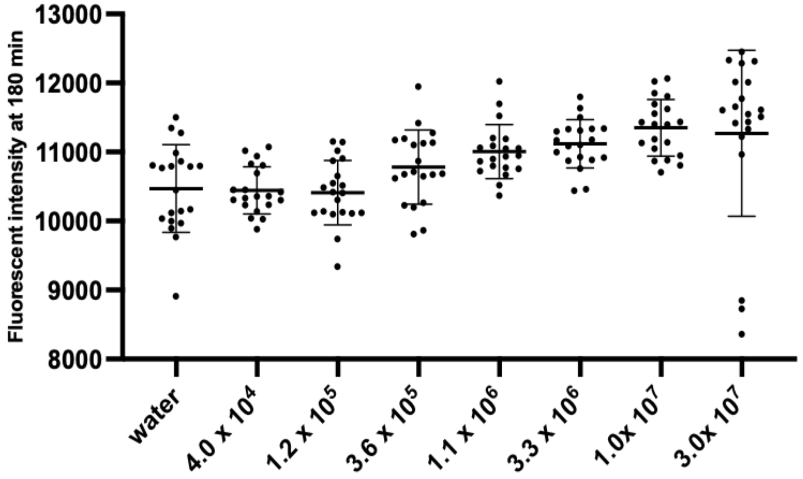

Figure S2. Determining the LOD for IAV cRNA-specific assay. 3-fold dilution series to determine the limit of detection. Each point represents one technical replicate (n = 20). The mean along with the standard deviation is shown.

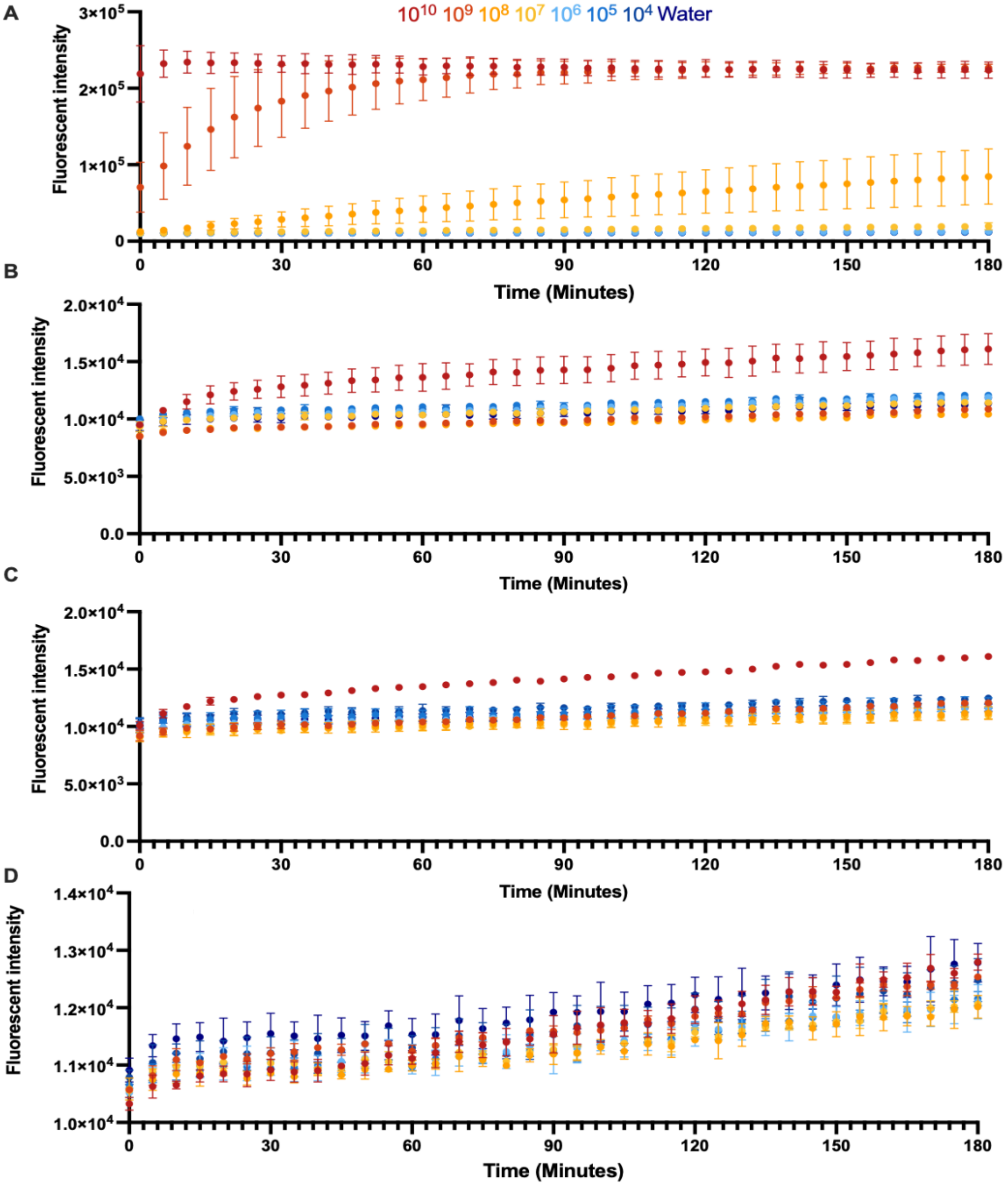

Figure S3. Cas13 detection of IAV Segment 5 vRNA. Detection of segment 5 vRNA from (A) A/WSN/1933 (H1N1) (B) A/Brisbane/59/07 (H1N1) (B) A/Oklahoma/05 (H3N2) (D) A/Vietnam/1203/04 (H5N1). The Cas13 fluorescent intensity vs time is plotted. Data was collected every 5 minutes for 180 minutes. Each point represents the mean of technical duplicates and error bars indicate the standard deviation.

Table S1. crRNAs used in the study

Name	Target	Sequence
TULV A	TULV S Segment (v-sense)	GACCACCCCAAAAAUGAAGGGGACUAAAACGUCAACUAGAGGGAGGCAAACAAUCAAG
TULV B	TULV S Segment (v-sense)	GACCACCCCAAAAAUGAAGGGGACUAAAACUGGCAGAGUUGGGAGCAUUUUUUUCCAU
TULV C	TULV S Segment (v-sense)	GACCACCCCAAAAAUGAAGGGGACUAAAACAACTAGAGGGAGGCAAACAATCAAGGAA
ANDV A	ANDV S Segment (v-sense)	GACCACCCCAAAAAUGAAGGGGACUAAAACGCAGUUAAUGGGGUUGGUUACUAACUAU
ANDV B	ANDV S Segment (v-sense)	GACCACCCCAAAAAUGAAGGGGACUAAAACGACUGCAGAAGAGAAGCUAAAGAAAAAA
ANDV C	ANDV S Segment (v-sense)	GACCACCCCAAAAAUGAAGGGGACUAAAACTTAATGGGGTTGGTTACTAACTATGGGT
IAV C A	IAV Segment 5 (c-sense)	GACCACCCCAAAAAUGAAGGGGACUAAAACGAUGGAAUUGGACGAUUCUACAUCCAAA
IAV C B	IAV Segment 5 (c-sense)	GACCACCCCAAAAAUGAAGGGGACUAAAACACGAUGCAACGGCUGGUCUGACUCACAU
IAV C C	IAV Segment 5 (c-sense)	GACCACCCCAAAAAUGAAGGGGACUAAAACCUAUGGAAUCAAGUACCCUUGAACUGAG
IAV V A	IAV Segment 5 (v-sense)	GACCACCCCAAAAAUGAAGGGGACUAAAACAGAUAAUCACUCACAGAGUGACAUCGAA
IAV V B	IAV Segment 5 (v-sense)	GACCACCCCAAAAAUGAAGGGGACUAAAACUUGGUGCCUUUGGUCGCCAUGAUUUCGA

Table S2. Clinical samples used in the study

Identity	Internal Code	Age	Sex	qPCR Value	Collection location (H1 or H3)	c-sense positive?	v-sense positive?*
c1	1644	22	F	19	ER (H3)	Yes	Yes
c2	1697 (47)	59	F	18	Unknown (H3)	Yes	Yes
c3	1703	26	F	25	ER (H1)	No	No
c4	1643	21	M	21	ER (H1)	Yes	Yes
c5	1683	2	M	23	ER (H3)	Yes	Yes
c6	1648	56	M	27	ER (H1)	Yes	Yes
c7	1690	12	F	19	ER (H3)	No	No
c8	1665	42	F	18	ER (H1)	Yes	Yes
c9	1645	4	M	11	ER (H3)	Yes	Yes (higher)
c10	1694	60	M	14	ER(H3)	Yes	Yes
c11	1681	25	F	29	D4 Ward (Death, H1)	Yes	Yes
c12	1732	92	M	12	ER (Death, H3)	Yes	Yes (higher)
c13	1701	48	F	23	ER (H1)	Yes	Yes
c14	1735	28	F	18	ER (H3)	Yes	Yes
c15	1704	94	F	30	L4 Ward (Death, H1)	Yes	No
c16	1688	18	M	15	ER (H3)	No	No
c17	1657	4	M	18	ER (H1)	Yes	Yes (higher)
c18	1682	83	M	26	PM Sample (Death, H3)	Yes	No
c19	1700	1	F	19	ER (H1)	Yes	Yes
c20	1722	32	F	19	ER (H1)	Yes	Yes

* (higher) indicates that the v-sense signal is higher than the c-sense signal.

Table S3. Primers used for in vitro transcriptions

Name	Description	Sequence
T7min_FW	IAV segment PCR for IVT	TAATACGACTCACTATAGGGTTATTAGTAGAAACAAGG
T7min_RV	IAV segment PCR for IVT	GCCGGCCAGCAAAAGCA
ANDV_S_T7_Add_Fwd	ANDV S_Segment PCR for IVT	TAATACGACTCACTATAGGGTAGTAGTATGCTCCTTGAAAAGC
ANDV_S_Rev	ANDV S_Segment PCR for IVT	TAGTAGTAGACTCCTTGAGAAGCTA
TULV_S_T7_Add_Fwd	TULV S_Segment PCR for IVT	TAATACGACTCACTATAGGGGCCGCCGGGTTATT
TULV_ST7_Rev	TULV S_Segment PCR for IVT	GGGACCATGCCGGCCTAGT
